# Coping and end-of-life decision-making in ALS: A qualitative interview study

**DOI:** 10.1371/journal.pone.0306102

**Published:** 2024-06-26

**Authors:** Celia Spoden, Olga Wenzel, Anke Erdmann, Gerald Neitzke, Irene Hirschberg

**Affiliations:** 1 Institute for Ethics, History and Philosophy of Medicine, Hannover Medical School, Hannover, Germany; 2 German Institute for Japanese Studies, Tokyo, Japan; 3 Institute for Experimental Medicine, Medical Ethics Working Group, Kiel University, Kiel, Germany; University of Auckland, NEW ZEALAND

## Abstract

How do people with amyotrophic lateral sclerosis (PALS) deal with their diagnosis and engage in end-of-life decision-making? What informational or supportive needs do they have for counselling about life-sustaining treatment and end-of-life care? Which correlating conditions and influences relate to these needs and how do they connect to the wish to die or wish to live? We conducted a qualitative interview study with 13 people with ALS in Germany from March 2019 to April 2021. Data collection and analysis followed a grounded theory-based approach and revealed close relationships between coping, informational needs and the preparedness for decision-making. We identified the coping strategies ‘avoid thinking about end-of-life’ and its counterpart, ‘planning ahead to be well-prepared,’ and differentiated the latter into the patterns ‘withdrawing from life and taking precautions against life-prolongation’ and ‘searching for a new meaning in life and preparing for life-sustaining treatment’. The approaches are based on individual perceptions, attitudes and motives and can be positively/negatively reinforced by healthcare professionals (HCP), family and other interpersonal networks, but also by disease progression and in reaction to health care services. Type and degree of needs concerning information and counselling differed according to coping strategies. These strategies may vary over time, resulting in different support needs. Our findings signify that deep insight is needed into PALS‘ coping processes to understand their decision-making about life-sustaining treatment. Healthcare professionals should be sensitive to illness experiences beyond medical aspects and foster coping as a biographical process to better support people with ALS.

## Introduction

People who receive the diagnosis amyotrophic lateral sclerosis (ALS) commonly react with shock, disbelief and dismay. They have just learned that their condition is incurable and average life-expectancy is two to four years [1], unless they opt for life-sustaining treatments like artificial nutrition (via percutaneous endoscopic gastrostomy, PEG) and non-invasive ventilation (NIV) or tracheostomy with invasive ventilation (TIV). People with ALS (PALS) face gradual loss of motor function, muscle weakness and difficulties in swallowing, speaking and breathing. However, there is a great variety in adjustment, which is explained by individual factors and how PALS cope with the diagnosis and integrate the illness into their lives [2].

We conducted a broader qualitative interview study on how PALS and their relatives engage in decision-making and on their wishes and needs for information and counselling about life-sustaining treatment and options of hastening death. The aim of our study was to develop a theory about PALS’ perceptions and requirements concerning information and decision-making support, how this is embedded into their illness trajectories, and related to specific coping strategies.

Most studies on coping with ALS are based on a psychological stress-coping model [3]. In this model psychosocial adjustment is understood as a complex interaction between illness parameters, cognitive appraisal, coping resources (personal characteristics, self-efficacy, health and social support) and coping strategies [2, 4]. The outcome of coping is measured as an affective state—most prominently the level of depression, anxiety and hopelessness—and quality of life (QoL) [4, 5]. Several studies have shown that coping strategies impact QoL [4, 6–8] and resilience [2], and that psychological status relates to physical decline and survival [8–10].

From a social-interactionist viewpoint, the psychological concept of stress and coping has been criticised and understanding chronic illness as biographical processes surrounding illness trajectories has been proposed [11, 12]. Corbin and Strauss have emphasised the social dimension of the illness experience, a sensitivity to the ill person’s experiences of bodily limitations and how these affect their self-concept and biographical processes [11]. The ill person has to deal with a triadic relationship of body, self and environment while incorporating the illness into their biography, coping with bodily and activity limitations, reconceptualising identity in terms of a new scope of performance and recasting new future perspectives into their biography [11]. Managing chronic illness is an ongoing process that involves the ill person’s biographical work, and work and care by their families and healthcare professionals (HCP) [11, 13]. For contextualising our analysis of how wishes and needs concerning information and counselling about life-sustaining treatment and options of hastening death are embedded in specific coping strategies, both perspectives—the psychological stress-coping model and the social-interactionist emphasis on illness trajectories—provide fruitful insights to understand coping in ALS.

## Methods

### Study design

We applied a grounded theory-based approach [14], since we aimed at studying the social interactions and experiences of PALS in the process of decision-making [15] on life-sustaining treatment and end-of-life options. Therefore, we conducted qualitative semi-structured open-ended interviews. The design was chosen to facilitate a deep understanding of how PALS deal with decision-making on life-sustaining treatment, what needs and wishes they have concerning information and counselling, and how they communicate about their wishes to live and die against the backdrop of their broader illness experience and coping.

For reporting the study, we used the consolidated criteria for reporting qualitative research (COREQ) checklist [16] as orientation.

### Research team, ethical approval, recruitment and sampling

Our study was based at the Institute for Ethics, History and Philosophy of Medicine at Hannover Medical School in Germany and was approved by the local ethics review board (No. 8148_BO_K_2018). The five study team members came from different disciplinary backgrounds, including social sciences, philosophy, public health, nursing and medicine (PhD, MA, MPH, MD), with a focus on ethical issues. When recruiting our study participants, we positioned ourselves as non-clinical researchers and not affiliated with an ALS clinic, to enable our participants to talk freely about their clinical experiences and to make clear that we would not provide medical counselling.

We recruited PALS through different channels: (1) since we wanted participants from all over Germany, we cooperated with several nationwide operating ALS support groups to spread our call via their homepages and newsletters; (2) we visited local support group meetings and presented our study or gave talks at informational events for PALS; (3) and we had a call for participants on our study homepage. It was important to us that the initiative to participate came from the participants themselves, since we strived to address sensitive topics as personal illness experiences, perception on quality of life, death and dying. The inclusion criteria were: (1) adults (aged ≥18), (2) with a confirmed ALS diagnosis, (3) sufficient German language proficiency, (4) treatment in Germany—as availability of and legislation on life-sustaining and life-shortening options influence the experiences and perceived options of participants–, (5) and providing contact details of a confidant in case further emotional support after the interview was needed. We also included PALS who were no longer able to speak verbatim and let them choose their preferred way of communication, such as communication aids, and interviews via email. No further explicit exclusion criteria were defined.

As part of theoretical sampling, we sought to maximise variability among participants with respect to age, sex, severity of disabilities, family status, educational background, economic situation and place of treatment. We strived for theoretical saturation; however, we might not have been able to include the most vulnerable PALS with fewer resources in terms of social support, socioeconomic situation, time and energy or with a fast-progressing illness (see limitations). We obtained participants’ demographic data during the recruitment process and from the informed consent forms and additional information during the interviews (Table 2).

### Data collection

We informed PALS interested in study participation about the aims, benefits, possible risks, voluntary participation, privacy and the study procedure. After the participants provided written informed consent, one of the researchers with experiences in qualitative research (either 1, 3 or 5) conducted an open-ended, semi-structured interview. The interviews took place from March 2019 to April 2021 at the participants’ place of choice, mostly at PALS’ homes. Two participants requested e-mail interviews due to speech impairments. During the COVID-19 pandemic we also offered interviews via video-chat (Table 1). We used an interview guide (S1 Table) based on our literature review [17, 18], which contained the following themes: 1) present life with the illness, 2) past illness experiences, 3) future plans and 4) informational and counselling needs for life-sustaining treatment, decision-making and options regarding death. The interviews were audio-recorded and transcribed verbatim, transcripts were shared with participants upon request. All names were changed into pseudonyms. Interviews were conducted in German, and quotes presented in this article have been translated from German to English by the authors. The number behind the name refers to the interview-sequence.

**Table 1:** Interview setting, duration and length.

Pseudonym	Interview mode	Interview supported by communication aid	Interview before or during COVID-19 pandemic	Interview duration [minutes]	Length of transcript [word counts]
David	face-to-face		before	115	20,196
Luise	face-to-face	yes	before	70	5,707
Bernd	face-to-face	yes	before	93	6,409
Andy	face-to-face		before	103	11,531
Paul	face-to-face		before	101	16,355
Sara	face-to-face		before	65	10,544
Julia	face-to-face		before	142	12,040
Frank	face-to-face		before	145	26,953
Chris	e-mail		before	(via 2 days)	3,548
Maria	e-mail		during	(via 3 days)	4,695
Tom	face-to-face	yes	during	170	4,362
Eva	video chat		during	68	10,821
Anne	video chat	yes	during	199 (on 3 days)	7,894

### Data analysis

Following a grounded theory approach [14], data analysis started during the data collection process to guide theoretical sampling. Three authors (1, 2, 5) coded in an iterative process the pseudonymised transcripts using the software MAXQDA. The interviews were first coded by author 1, codings then reviewed by authors 2 and 5, and emerging categories discussed with the whole team. Via open coding, we generated inductive codes, which we developed further into categories. During axial coding, we identified conditions which caused the particular phenomenon, defined category properties, explored actors’ (inter)actions and strategies, and the resulting consequences. In the selective coding process, we further related and integrated the categories. We constantly revised our code structure, developed hypothesis from the data and tested our theoretical ideas against the empirical data. All authors took part in discussing the findings.

## Results

### Interview characteristics

The interviews lasted between 65 to 199 minutes. However, the duration does not necessarily correlate with the length of the interview transcript, as impairment of speech or communication aids slowed down the speed of communication. At the request of the participants, two email interviews and one video chat interview were divided into several sessions (Table 1).

### Study population

We interviewed 13 participants, six women and seven men. Participants’ ages ranged from 46 to 80 at the time of the interview, and from 37 to 78 at the time of diagnosis. The time between diagnosis and interview ranged from five months to 201 months. Almost all participants used a wheelchair (11), five had PEG, three used NIV and two lived with TIV. The onset was bulbar and spinal in six cases respectively, and unclear in one case. Two PALS had the familial form, but only one of them experienced the disease in relatives directly before being diagnosed herself. Most of them were cared for at home by their relatives. Five used communication aids (Table 2).

**Table 2:** Characteristics of interviewed PALS.

**Gender**	
female	6
male	7
**Age at the time of interview (Age at diagnosis)**	
30–39	0 (1)
40–49	2 (1)
50–59	5 (7)
60–69	3 (2)
70–79	2 (2)
80–89	1 (0)
**Family situation**	
married or with a partner	11
single	2
with children	11
**Living situation**	
with a partner	10
with a sibling	1
in a care facility	2
**ALS form**	
bulbar	6
spinal	6
unclear	1
of which familial form	2
**Disease duration** (time between diagnosis and interview)	
< 1 year	4
1 < 3 years	2
3 < 5 years	2
5 < 7 years	3
12 years	1
16 years	1
**Used aids**	
wheelchair	11
percutaneous endoscopic gastrostomy (PEG)	5
non-invasive ventilation (NIV)	3
tracheostomy and invasive ventilation (TIV)	2
communication aids	5

### Analysis of interviews

**Fig 1:**
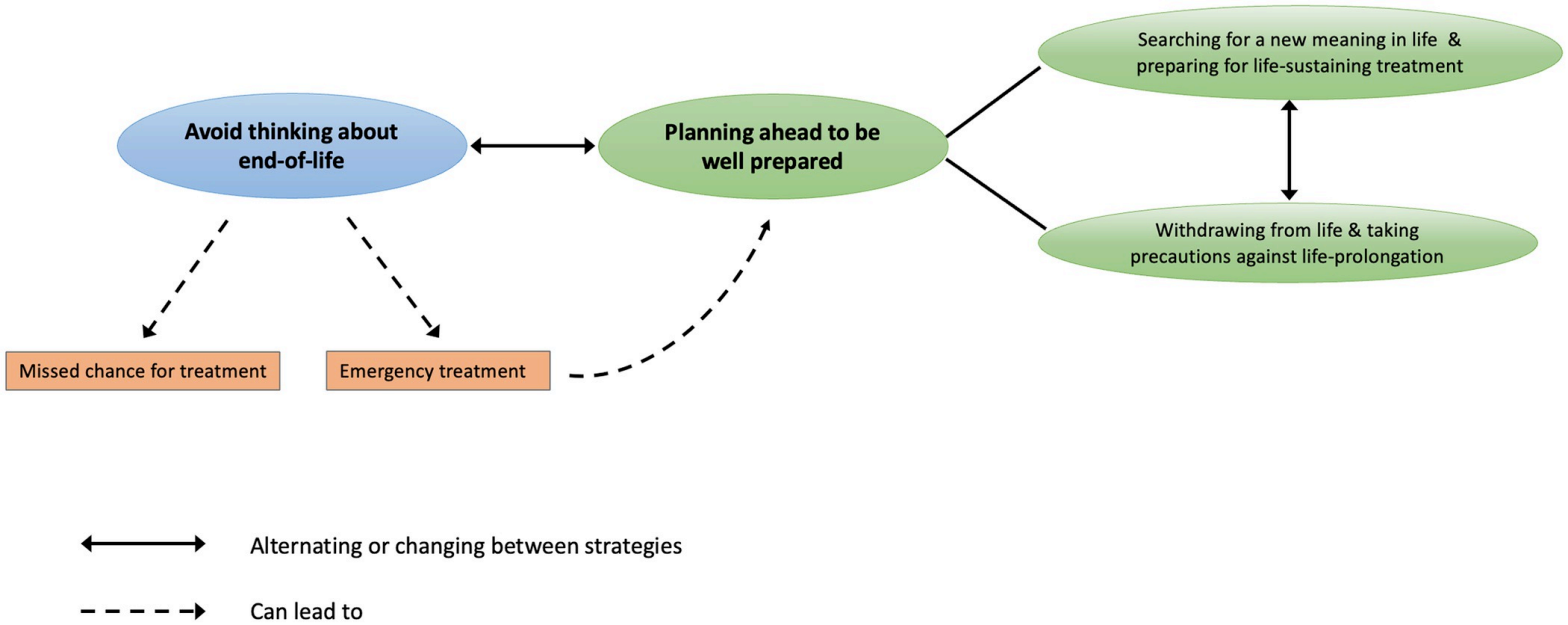
Coping strategies.

The analysis of our empirical data revealed how the wishes and needs of PALS regarding counselling about life-sustaining treatment and end-of-life care are embedded in specific coping strategies, which we present here. From the analysis, two core categories emerged which distinguish our participants’ approaches towards their illness in ‘avoid thinking about end-of-life’ and its counterpart ‘planning ahead to be well-prepared’. The latter can be differentiated into the patterns ‘withdrawing from life and taking precautions against life-prolongation’ and ‘searching for a new meaning in life and preparing for life-sustaining treatment’ (Fig 1). PALS’ different approaches are based on their individual perceptions, attitudes and motives and positively or negatively reinforced by HCP, family and other interpersonal networks, support groups, healthcare system experiences and progression of their disease. The strategies are not exclusive. Some PALS employ different strategies simultaneously, alternate between strategies or change from one to another over time.

#### Avoid thinking about end-of-life

An ALS diagnosis and the accompanying prognosis often destroy future perspectives and cause fears and feelings of hopelessness. Participants refer to negative thoughts as ‘dark moments’ that make them feel incapable of acting in everyday life. One strategy of dealing with these dark moments is: avoid thinking about end-of-life or future scenarios.

‘*I’m still alive*, *but there are also phases where I have the feeling that I’m completely lacking perspective*. *I always try not to stare at the end like a rabbit at a snake*. *Rather*, *I’ll say*, *I play it by ear from one day to the next*. *And see how I get along*. *But there are also very dark moments*, *yes*, *with the perspective*’.

(Paul: 7)

Some PALS avoid support group meetings because this confronts them with later disease stages. In the extreme, PALS close their eyes to reality, do not want to know and ignore provided information. They do not consider the upcoming consequences of physical decline, prepare countermeasures or engage in decision-making on PEG, NIV and TIV.

If they put off dealing with the disease for too long or progression is fast, PALS risk missing the right timing for making important decisions. This can mean it is too late for PEG or, in case of respiratory insufficiency, that others must make an emergency decision. If physicians or family decide in favour of life-sustaining treatment, PALS must cope with this decision afterwards. They either accept the decision or struggle with it and demand the discontinuation of treatment. Participant Chris ignored all information after diagnosis and ended up in an emergency situation. His wife decided for NIV in his place and one year later, in a second emergency for TIV:

‘*I had no information about mask ventilation*. *This took place only three weeks after the diagnosis*. *My wife was very concerned*, *read a lot about the disease and knew that I suffered from CO2 poisoning*. *[…] I closed my eyes to reality and did not want to know anything*. *But the mask helped me and I could accept it*. *[…] I am very grateful to my wife for deciding instead of me*. *[…] I ignored everything about my future*. *I didn’t want to talk about my end*. *Did not ask any questions*’.

(Chris: 65, 78, 79)

Finally, Chris has come to terms with his illness and future decision-making. In retrospect, he emphasises how important it is for physicians to provide comprehensive information about the disease and its consequences from the moment of diagnosis and continuously thereafter, as PALS do not want to hear and deal with this at the beginning.

However, avoiding or postponing decisions or specific thoughts does not only occur in early stages of the disease. Participant Andy represses thoughts on the time when his NIV will not be sufficient anymore because it is anxiety-ridden. Another participant, Tom, has been living with TIV for ten years. He sometimes worries about the end-of-life, but tries to put the topic aside.

Since there is no cure for ALS in conventional medicine, some PALS search beyond it for alternatives. These PALS pursue hopes for a treatment or cure and try complementary treatment to conventional medicine. Yet, the belief in a cure can also function as ‘avoidance’ of thinking about the end-of-life, since PALS hope or believe that they are not going to die of ALS and perceive no need for information or counselling. Ann for example does not think about end-of-life matters and explains: *“I am trying many alternative treatments and have signs that I won’t die of ALS*.*”* (Ann: 100).

Ignoring the illness and putting-off decision-making means giving up control. Realising this can be a turning point and foster a more active strategy of planning ahead to be in control of the future.

‘*Sometimes I feel like I don’t really want to know*. *I prefer to go day to day*. *And then*, *however*, *THAT also breaks down again*. *And I think*, *it doesn’t work like that*. *I must look much further into the future*. *Maybe we must modify more [in our house]*. *Or*, *because I don’t want to go to a nursing home*, *must I prepare for that*? *Oh*, *that’s all very difficult’*.

(Paul: 11)

Some PALS alternate between avoiding and planning ahead. For them, avoidance has the function of not getting lost in negative thoughts and feelings.

#### Planning ahead to be well-prepared

Another strategy is to plan ahead, organise and take precautions for future stages of the disease in order to be well-prepared. Although transitions are gradual and some PALS alternate between both strategies, planning ahead is based on wanting to know about the disease and what options there are. It is associated with seeking control over life and not wanting to be surprised by sudden functional decline when assistive devices are not yet in place. Maintaining independence, autonomy and privacy are strong motivations to engage in planning ahead. PALS observe their body and functional changes in an attempt to estimate the course of the disease. However, uncertainties of progression rate make it difficult to anticipate what symptoms are coming next and in what order. This makes adaptation, prioritisation of tasks and planning ahead a challenge. Furthermore, the challenge of planning ahead is increased by fast symptom progression, being solely alone in charge of planning and organising, or a lack of support by HCP. Sara explains:

‘*I just want to have a certain amount of time for preparations*. *I think the doctors should actually point this out to you*. *Because they know what’s coming*. *[…] But no doctor talks about that*. *I’ve figured it all out for myself*, *because I know what’s coming*, *I’m prepared*. *But nothing comes from the doctors*, *nothing whatsoever*’.

*(Sara: 119)*

The actions PALS pursue to (re-)organise their lives with the disease and plan ahead are manifold: They seek information about the disease, medical options, including medical trial participation, gene-therapy, and complementary or alternative treatment. Some follow ALS-research, hoping for development of a cure or new treatment and discuss their findings with their physicians. Concurrently, they inform themselves about advance decision-making including advance directives or options to hasten death. Working their way through the healthcare, social care and benefits systems is time consuming and sometimes frustrating.

Organising future living arrangements, care and aids are demanding tasks. Sooner or later, falling becomes a safety matter. Some PALS consider moving to a care facility. PALS who want to stay home must make their homes barrier-free and organise home care. Organising home care brings experiences or anxieties about nursing staff shortages that can be life-threatening and lead to existential fears.

‘*I have a perfect environment here*. *My wife stands by me*, *that’s the most important thing*. *But my biggest concern is care services*. *There are no nurses*. *And my careservice is very small*. *If one of them [a caregiver] drops out*, *or is ill*, *then we are faced with chaos*. *[*…*] I’m afraid*. *Because without care I can’t exist*’.

*(Andy: 98)*

Planning ahead is the opposite of to avoid thinking about end-of-life. However, if PALS feel overwhelmed by thoughts on future prospects and perceive too many tasks, they might alternate between planning and avoidance to protect themselves from getting lost in depressive thoughts. Additionally, the planning ahead strategy can be differentiated into the patterns ‘withdrawing from life and taking precautions against life-prolongation’ and ‘searching for a new meaning in life and preparing for life-sustaining treatment’. The differentiation depends on whether a PALS perceives life as more and more meaningless and plans ahead to make sure they do not live longer than they can endure life, or if they find new meaning in life and plan ahead to stay alive for certain events or for certain reasons.

### Withdrawing from life and taking precautions against life-prolongation

Diagnosis and prognosis can destroy future perspectives to an extent that a meaningful life seems to be already over. Shortly after diagnosis, some participants report being suicidal. Some denounce any future plans, locate the meaning of their lives in the past and give up hobbies and interests. Some can maintain or even intensify social relationships, which become a source of meaning and strength and help them endure life with ALS. Others withdraw from social relations and do not want to burden anyone with their illness or negative thoughts. Cutting-down social interactions is also an attempt to regain control by limiting life to a small manageable area:

‘*This time*, *when I still had enjoyable experiences*, *is getting further and further away*. *Because*, *you must deal a lot with this illness*, *and not only coping with the illness*, *but with all the aids and insurance companies and so on*. *[*…*] That is not beneficial*. *Now*, *I have little to no more positive experiences*. *And that upsets me a bit*. *Even though I said I don’t want to do that [hobbies] anymore and I don’t want to go there anymore*, *which under the circumstances*, *I could certainly still do*. *But that is somehow too much effort*, *because*, *as I just said*, *I need my easy-to-control area’*.

(David: 323–325)

Additional factors also intensify withdrawal from social life: Family and friends might have difficulties coping with the disease and conflicts occur or family/friends stay away. With decreasing mobility and ability to verbally communicate, leaving the house and interacting becomes more exhausting and challenging, especially if aids, barrier-free environment, and/or supportive social services are lacking. PALS experience applying for aids, filing claims against claim denial, and struggling with healthcare and long-term care insurers as exhausting, humiliating and as a race against precious lifetime. This deprives PALS of energy for meaningful activities and interactions. Singles seem even more vulnerable to social withdrawal and isolation, because they must shoulder everything on their own. If they stop pursuing hobbies and engaging in meaningful relationships beyond the disease, PALS become absorbed in disease-related activities and have no positive experiences that balance negative ones.

These PALS think they have no choice in the present other than accepting help and adjusting their daily lives to the illness. They have the feeling of running after the disease and of being just in time or too late with adapting their everyday life. Consequently, they have the impression of being in an ongoing adaptation process, comparable to a downward spiral that never reaches an end-point where they can come to rest. In their perception, meaning and QoL losses correspond to functional decline: the more functional limitations progress, the more they perceive meaningful life as fading away.

‘*It has already started [to get difficult]*: *Pouring a drink*, *going to the toilet*. *[…] It’s getting harder day by day*. *[…] At the MOMENT it still works*, *but at some point*, *it just won’t work any longer*. *So*, *life decays with the body*. *From week to week something crumbles away somehow’*.

(Sara: 49)

Some struggle to accept the illness, quarrel with fate and ask ‘why me?’. They mourn their lost abilities and hobbies or interpret functional decline as their body and former self dying. Some try to adapt their hobbies, but this only seems possible to a certain degree and they do not actively search for new meaningful activities. The perception is that the disease limits the remaining lifetime and death is already approaching. For PALS who do not regain meaningful activities in the present, life with ALS becomes a persistent state of enduring.

Since they perceive that QoL corresponds to physical functioning and consequently declines with physical deterioration, they fear experiencing a helpless period of suffering before natural death occurs. They reject life-sustaining treatment from the beginning because accepting it would prolong a life that is already difficult to endure. Most PALS have not been informed about life-sustaining treatment options by their physicians before deciding on rejection and do not perceive a need to discuss their decision. However, they consider treatments such as NIV or PEG when implemented as symptom control or QoL improvement.

Although some PALS recognised that they can endure more than they had thought previously, they fear a point in the future when they cannot endure life any longer. For this reason, they take active precautions against life-prolongation. Yet, beyond rejecting life-sustaining treatment, they want to have the option of ending their lives in a peaceful way, preferably alone at their home. In their imagination, having a lethal drug at home for a future suicide would make them feel secure. In addition, they imagine having this fatal drug would enable them to endure life longer.

‘*I am afraid of suffocating*. *That’s cruel*. *I’m not afraid of death*. *But of dying*. *In such a cruel way*. *That…it would be a reassurance to me to know that then*, *when it can no longer be avoided*, *that I can then end it*. *[…] I imagine that if I knew I had a pill in my drawer with which I could end it all immediately that would calm me down*. *This doesn’t mean that I have to use it*. *But just having the certainty that I could*, *that’s it ‘*.

(Andy: 3–7)

Fear of a ‘bad death’ is sometimes based on the idea of having to suffocate in agony, when PALS have not been informed about palliative measures such as sedation, or if they do not believe in the effectiveness of palliative measures.

In other cases, PALS interpret the PEG as a sign of the approaching end and have no information on the PEG as symptom control and QoL improvement, or the possibility to discontinue treatment. For example, Paul, whose hypothetical wish to die is based on the idea that he will end-up completely immobilised, hooked up to machines and helplessly waiting for death to occur, asked his doctor if there was a pill to end life quickly. Instead of asking Paul what was behind the wish to hasten death, his physician dismissed his query by saying that Germany prohibits euthanasia. There was no conversation about palliative care options or being able to discontinue life-sustaining treatment. Paul was left alone with his fears.

### Searching for a new meaning in life and preparing for life-sustaining treatment

For some PALS, the lack of perspective in which life no longer makes sense and the disease seemingly takes over control, is a phase that follows the shocks of the diagnosis and prognosis, but which they do overcome.

‘*When I got the diagnosis*, *there were three options for me*: *Jump right off the skyscraper*. *No*, *Frank*, *you*, *the hero of everyday life […]*. *End up like that*? *No*. *I’ll jump from the skyscraper…or sit down in the corner at home and mope*. *But neither would have suited me*. *[…] I like living too much for that*. *Have I already mentioned that one wants to live in spite of everything*? *Moping around*, *sitting in the corner*? *Me*? *Too boring*. *So*, *let’s make the best of it*. *Yes*, *that’s what I try to do*.’

(Frank: 213)

PALS that exhibit the ‘searching for a new meaning in life and preparing for life-sustaining treatment’ strategy do not let the disease take over their life. They accept the illness and make it one part of their life next to others, ‘*I live with the illness and not for the illness*,’ as Eva (109) puts it. Despite the fatal diagnosis, such PALS still perceive they have a choice about how to carry on living and decide to pursue their hobbies and interests and engage in precious relationships with family and friends.

‘*I deal with it; I don’t get into it or anything… I mean*, *I still have another life*. *I’ve always said*, *the illness has arrived in my life*, *it’s there now*, *I didn’t ask it to come*, *I can’t kick it out*, *so I must come to terms with it somehow*. *I must cope with it and it is there*. *I don’t like it*, *it can sit in the corner*, *but it’s there*. *And every now and then I look after it*. *(Laughs*)’

(Frank: 161)

Some put the fatal diagnosis into perspective by considering that everyone will die someday and whether or not you have an incurable, fatal disease and think you know how much time is left to live, death can come calling any time. Others deny their physicians the power of defining their lifetime with prognoses and counter it with a positive attitude towards life.

‘*I do not allow myself to be frightened*. *And when I was told*, *without requesting it*, *how long I had left to live when I was diagnosed*, *I immediately said that my life expectancy would double and that’s only because of my positive inner attitude*. *I am now in my eighth year of living with ALS* ‘.

(Maria: 29)

These PALS must still say goodbye to some hobbies because of somatic progression, but instead of mourning, they perceive it is more important to enjoy what they can still do. They preserve their humour and question social expectations of the role of the ill. They reflect on existential questions such as if ‘just being there’ in a completely locked-in state can still be meaningful or if discontinuing ventilation is in accordance with their faith. Spending time on meaningful activities and relationships is important. This means that these PALS do not let the disease dominate their daily lives. They plan ahead, organise aids, go to prescribed therapies and appointments in the clinic, yet are concerned about a balance between these disease-related necessities and a life beyond the illness. After all, hobbies and interests and maintaining personal relations make them feel alive and give their individual lives meaning. Some write down their life-story or find pleasure in passing on knowledge and experiences to children and grandchildren. Julia emphasises the importance of psychotherapy where she can openly address her concerns without worrying about burdening her family with her thoughts.

‘*This also affects the relatives*. *My daughter […] also likes to have the frankness*, *but I sometimes wonder if that’s good for her*. *And that’s why it’s important that when people want it*, *they get therapeutic support… Because*, *that’s from one day to the next*, *you first see the finiteness [of your life]*. *How are you supposed to cope with all that in such a short time*? *Yes*, *it’s nothing earth-shattering*, *but it gives me security that I can address certain things that I observe in myself*.’

(Julia: 47)

For these PALS, life still has a future perspective and they pursue or live for goals. Most of them are open to life-sustaining treatment and are willing to try things out. Since they experienced being able to adapt to their illness and perceive it as difficult to anticipate the impact of life-sustaining treatment on their life, they adopt step-by-step decision-making according to their QoL. Likewise, they consider continuous and step-by-step consultation as important, where counselling stays one step ahead of the disease. The present, subjective QoL and their families’ wellbeing are key decision-making criteria. They feel that many give up too quickly and that it is wrong to reject everything from the onset. In their opinion it is important that support groups, friends and family convince PALS to improve their present situation and QoL. This also means trying out a wheelchair and a PEG, which primarily improve current QoL.

For PALS following this strategy, meaning in life and QoL are not necessarily correspondent to physical functioning. They perceive that a meaningful, good QoL is possible with NIV, PEG and for some also TIV. They trust their physicians and (palliative) medicine that something can be done to ease pain and suffering and that there is no need for a lethal pill. These PALS imagine, if they have the wish to hasten death, they will be using life-sustaining treatment which could be discontinued on request:

‘*Well*, *I think that if I’m in SUCH a bad condition that I’d think [of not wanting to live any more]*, *this would definitely be a situation in which I had some kind of life-sustaining treatment*. *Because you can do something about everything else*. *And then I’d have them [any life-sustaining treatment] turned off*. *Then I needn’t take the pill*. *[*…*] That [taking a lethal pill] is somehow out of the question for me*, *from a religious point of view ‘*.

(Eva: 103)

Deciding step-by-step is also a strategy to reduce the complexity of the present situation and not be overwhelmed with decision-making. These PALS prefer a wide range of potential choices for action. They want to be well-informed about all options, even trying out TIV and then discontinuing it should they or their family not be able to adapt to the situation. In this sense, they do not perceive decisions as final, but as a process in which they can adapt and change flexibly according to their experiences, preferences and needs. Tom had pneumonia and decided for a tracheostomy in an emergency and continued living with the invasive ventilation later on to see his child grow up. He recalls that this decision was embedded in an adaptation process and that he could not even have imagined a wheelchair after diagnosis.

Deciding step-by-step can also mean PALS want the opportunity to decide whether to discontinue life-sustaining treatment depending on their current situation and QoL. Eva, who uses NIV, wants to decide everyday anew whether she wants further life-sustaining treatment or not. However, since communication is the prerequisite for this form of decision-making, she wants all life-sustaining treatment to be terminated once she loses her ability to communicate.

It is striking that most of the interviewees pursuing this strategy had already faced—and overcome—difficult life situations in the past. These fateful experiences range from the early death of a parent, child or partner, divorce, or diagnoses with other chronic illnesses. They learned that arguing with fate or complaining leads nowhere. Participants refer to their positive attitude towards life, support from family and friends, pursuing life goals, living for their children/family/dog, and their faith as sources of strength for coping with past and present difficult life situations.

## Discussion

The results show how PALS’ informational needs and wishes concerning life-sustaining treatment and dying are embedded into their coping with the illness. Here we discuss how this relates to the literature.

### Need for information and preparedness for decision-making

Whether or not and how much PALS want to know about the disease, progression and future possibilities depends on their coping style. Avoidance relates to a low self-perceived need for information and counselling. However, the professional perspective will likely associate this with a high information need. PALS who solely pursue this strategy do not want to know or be informed about life-sustaining treatment and their choices. This ultimately results in giving up control about decision-making and their future and risking a QoL decrease. In this sense avoidance exemplifies a maladaptive strategy.

Yet as we have shown, avoidance can also have a protective function in terms of emotional coping when used to distract from negative feelings and concentrate on the present to remain capable of acting. This coincides with findings from the literature [4, 19]. Matuz et al. [4] reported that minimising the importance of the diagnosis or directing attention away from negative information and consequences might be beneficial in early stages of the illness. Doing so may prevent PALS from experiencing psychological distress and despair and can result in higher QoL. As the disease progresses, though, avoidance can become a maladaptive strategy because it hinders further coping [4]. However, Young & McNicoll found that taking ‘one dose of reality’ at a time, or consciously blocking out negative thoughts as strategies of reframing and managing the emotional impact of the illness can also be beneficial in later stages [19]. Therefore, a combination of confronting and avoiding strategies might be beneficial if PALS engage in future care planning [4].

With our interviewees, avoidance became problematic when it was the only strategy used for too long, or if disease progression was fast, and the right time for decision-making was missed. When avoidance was used in combination with believing in a cure by alternative medicine, it potentially resulted in denying ALS fatality. In PALS without regular medical care and undiscovered misconceptions, avoidance can lead to (suffocation) anxieties. This in turn might motivate a hypothetical wish to hasten death.

From an ethical viewpoint, there is a right not to know—and the wish not to be informed should be respected. However, as avoidance is sometimes a reaction to the shocks of the diagnosis and the prognosis or results from anxieties or feelings of hopelessness [20], the wish not to be informed about future prospects should not hinder communication about illness experiences and concerns. HCP should listen to their patients, aim to detect misconceptions and fears, and address these adequately.

The literature reveals different findings about the need for support and information. Whereas some studies have emphasised a need for support and information during the early stages of the disase and after diagnosis [21–23], others highlighted the necessity of offering it repeatedly, even when it was frequently rejected at diagnosis [24]. Providing PALS with information when they ask for support in terms of aids and emotional comfort may also increase QoL [4, 22, 24]. Therefore, some authors have argued that communication and mobility difficulties likely limit social interaction and the social support resource [4, 24]. This coincides with our findings that obstacles in organising life with the illness have an exhausting, demotivating and humiliating effect. Furthermore, our participants who pursued the planning ahead strategy had a high demand for information about how to enhance well-being in the present, aids, precautionary treatment, support and care. They drew on several sources including physicians and physio, speech or occupational therapists at the ALS clinic, the internet and peer-support.

Concerning informational needs on life-sustaining treatment, there were great differences: PALS who withdrew from life and gave up the pursuit of a meaningful future did not want to prolong life and rejected life-sustaining treatment from the beginning, without being informed about those options by their physicians. They perceived the decision-making as final and complete and did not want to discuss their decision with anyone. PALS, though, who found new meaning and took back control over their lives had a high demand for information and counselling about starting and ending life-sustaining treatment (including TIV) and palliative care. These PALS did not want to make decisions in advance but with respect to their current QoL. Therefore, they adapted a step-by-step pattern of decision-making.

Some authors have argued [25, 26] that ALS’s progressive nature means coping with the disease and decision-making is not a linear process but rather cyclic. According to King et al., active coping strategies, sustained self-esteem, positive self-perceptions and being in control contributed to decreased stress levels and good wellbeing. In contrast, passive strategies led to lowered self-esteem, negative self-perceptions, feeling the disease controlled everyday-life, increased stress levels, and negatively-impacted wellbeing. They argued that there might be ‘windows of normality’ but unlike in other chronic diseases, periods of normalisation cannot be reached in ALS, as PALS are constantly challenged with ongoing changes and adaptation [25]. However, some of our participants shared that they live normal lives. This might relate to a low progression rate or to the fact that we included PALS with TIV—unlike King et al. [25]. TIV might allow for a normalisation process to take place in an advanced state of the illness. This is also supported by reports from Japan, where some PALS on TIV even return to a life of economic activity comparable to the general population [27]. Therefore, it is crucial for comprehensive care in ALS to repeatedly offer access to different sources of information, focus on maintaining interpersonal networks and provide PALS with readily available aids and adequate communicational devices that support PALS in maintaining an active role in their families and society [4, 24].

### Perception of choice and control, meaning in life and the wish to hasten death

As some studies [20, 28–30] have shown, the uncertainty about illness trajectory and loss of control is troublesome and doubts about their decisions lead PALS to change their minds [23]. However, the greatest difference between our study participants who withdrew from life and PALS who searched for new meaning is their perception of available choices and the relation between physical impairment and QoL.

As Detka showed in his study on chronic illness, individuals who center their lives on the illness and make it the fundamental relevance structure see themselves in a biographical ‘end position’ and set aside plans for the future [12]. This corresponds to PALS in our study, who withdrew from life and took precautions against life-prolongation. They tended to think there was no choice and doctors had nothing to offer, as there is no cure for ALS. For them, loss of meaning and QoL corresponded to functional decline. They perceived meaningful life as already or almost over, and death as approaching, so they did not actively search for new meaningful activities. Focussing predominantly on illness-management narrowed their perspectives and resulted in a feeling of rushing after the disease. Hope of having reached an acceptable situation was destroyed by further functional decline, resulting in a feeling of never-ending losses. This also led to the threatening feeling that with progression of physical limitations, control over life is further lost and dependence intensifies. Many of these PALS not only actively took precautions against life-prolongation, but also considered euthanasia or assisted suicide to avoid suffering. Palliative treatment was not perceived as a ‘true’ alternative but as having no other (legal) option. Ohnsorge et al. also reported that the hypothetical wish to die had the function of maintaining control and a sense of agency. Whereas becoming a member of a Swiss right-to-die organisation served in their study as a last resort [31], our participants rejected the option to travel to Switzerland, mostly because they wished to have ‘the pill’ at home and decide on a daily basis if life was still bearable. Continous, open conversations with physicians about options to die, life-sustaining treatment and palliative care are paramount for discussion on advance decision-making [24, 32] and have been reported to counteract life and death anxieties [20, 28] or the wish to die [31]. Kremeike et al. found that most palliative patients appreciated a proactive assessment of wishes to die. Their study emphasised that the desire to die may be a way to cope with illness and that proactively addressing the subject and allowing emotional expressions may be beneficial, foster a trusting relationship and even result in preserving the will to live [33]. This corresponds with our participants’ high demand to be informed about dying with ALS and discussing death.

In contrast to an illness-centered living arrangement with chronic illness, Detka reported a biographically-oriented living arrangement [12], which has many aspects in common with our strategy of searching for a new meaning in life and preparing for life-sustaining treatment. In this living arrangement, the illness is only one aspect existing alongside other relevant life aspects. It is integrated as one part of identity and the ill person engages in biographical planning and makes future plans again. With active coping strategies, they aim to limit the effects of the illness on everyday life and constantly adapt their coping. They preserve sources for a meaningful life and maintain biographical continuity [12]. This accords with studies that have shown it is not the illness itself that is a stressful or threatening event, but the way it is perceived, interpreted and judged by the individual [3, 8, 34].

Young & McNicoll reported that PALS who cope extraordinarily well distinguished between developments they could and could not control. They used problem-solving strategies for the former and decided to determine their emotions concerning the latter [19]. This corresponds with our finding that PALS who regained control and found new meaning consciously decide not to mourn lost physical functions and activities. They concentratde on activities they could still do. This is confirmed by studies which have shown that physical decline does not necessarily correspond to poorer subjective QoL or well-being [4–6, 35–39]. Whereas negative coping styles unfavourably affect QoL and well-being, positive or active coping styles may have a positive effect on both [4, 7, 8] or even on survival [8]. Pagnini et al. showed that mindfulness predicts higher QoL, lower depression and anxiety, and a slower physical decline which might lead to longer survival [10]. In fact, many mindfulness concept features correspond to the attitude, characteristics and actions of our participants who found new meaning and regained control. Mindfulness has also been reported to be a protective factor for family caregivers and their perception of burden, and positively correlates with their QoL [34, 40]. This is of utmost importance, since several studies have shown that feelings of being a burden may exacerbate PALS’ desire to hasten death [28, 29, 31, 41–43] or reject life-sustaining treatment [42]. This was true for our participants, regardless of the patterns of withdrawing from life or searching for new meaning. Conversely, studies have revealed that family and friends are the most frequently mentioned and beneficial source of strength and emotional support [20, 24], which corresponds with our results.

Religion and spirituality have often been mentioned as a source of well-being or protective factors against the wish to die [19, 24, 36, 43–45]. However, the importance of religion and spirituality seemingly depends on the sociocultural framework, since these factors were of minor importance in other studies [8, 20]. Some PALS in our study referred to their faith as a source of strength. Whereas for two of them euthanasia or (assisted) suicide was compatible with their religious beliefs, for two others it was not an option because of their faith.

Several studies have highlighted the need for continuous support throughout the illness process regarding existential questions of finding meaning in life and thoughts of death. For HCP, this requires focussing not only on disease symptoms but also on supporting the individual in finding adequate coping strategies and meaning in life, encouraging them to seek social support [4, 6, 20], and providing support in their decision-making process [23]. Hogden et al. [26] found that a specialised, multidisciplinary clinic can provide the best support and decision-making cannot be rushed, especially when PALS are overwhelmed and need time to accept the illness. Our findings add that some of our participants preferred talking with psychotherapists or clergy about existential questions, including deciding about life-sustaining treatment. Therefore, it might be beneficial to include staff trained in psychological, spiritual, religious or ethical counselling in the care team.

## Reflections on strengths and limitations

One strength of our study is that we included PALS with impaired communicational abilities who used communication aids. Since we did not recruit via a clinic, we also had participants who had more or less dropped out of medical care due to bad experiences. Our study included PALS in different stages of the disease and with a variety of physical symptoms. This helped us detect and consider different coping strategies and preparations for decision-making. However, as our study is not longitudinal, we can only refer to the changes and developments our participants reflected in their narrations.

We strived for theoretical saturation, however, the opportunities for theoretical sampling were limited. ALS is a rare disease and therefore the population of PALS is small. In addition, the progression of ALS varies and ALS is a time-consuming disease. As other studies reported, PALS might decline study participation because of a lack of energy [23]. We noticed, for example, that a high share of our participants were well-educated, financially well-off, and with a more or less functioning support network. This might have resulted in more available options or comparably high coping resources and more adaptive coping strategies. We assume that people with a fast illness progression, without a well-functioning support network, and in financially difficult situations did not have the resources to take part in our study or did not expect any benefit from participating. Therefore, and because PALS had to take the initiative and contact us about participation, we cannot rule out an incompleteness of coping strategies and a selection bias. Furthermore, we observed that some PALS participated in order to voice their resentment about the legal restrictions on euthanasia, or concerns on the care situation in Germany. In contrast, others intended to demonstrate that life with a disease as ALS and life-sustaining treatments can be meaningful. Therefore, our study participants might not represent the ALS population in Germany, but PALS with intense experiences or opinions. Regarding the applicability of our results to the global ALS population, there might be limitations in terms of the legal framework in Germany which allows treatment discontinuation but forbids euthanasia. Further limitations could be caused by Germany’s health and social care system and caregiver situation. Costs for PEG, NIV and TIV, and intensive and palliative care in general are covered, but the intensive care sector was undergoing changes during our study and is characterised by a nursing staff shortage.

## Conclusions

The results of our study have an impact on the professional medical encounter. With respect to their coping strategies, PALS have different needs for information and support, which may vary and develop over time. Our study reveals that understanding the coping strategies and biographical changes during the illness is a prerequisite for patient-centred counselling, supporting decisions about life-sustaining treatment and addressing attitudes towards options of hastening death. HCP should be sensitive to the illness experiences beyond physical or medical aspects of disease and foster coping as a biographical process to empower PALS and help them feel in control. This means creating ‘time-outs’ from the illness and its medical management and room for distraction and meaningful activities. In addition, psychological, spiritual or religious counselling may be beneficial and provide PALS with opportunities to voice their experiences, emotions and thoughts on living with the illness or reflect about existential issues such as a meaningful life or a good death. Furthermore, our results highlight the urgent need for improvements on a societal level. These include dismantling bureaucratic hurdles involved in applying for aids and assistive technologies, or addressing the nursing shortages, which affect PALS’ QoL or may impair PALS’ autonomy and existential needs.

## Supporting information

S1 Table Interview guide. (DOCX)
