# Supplementary material for: Coping and end-of-life decision-making in ALS: A qualitative interview study
Source: PLoS One. 2024 Jun 26;19(6):e0306102. doi: 10.1371/journal.pone.0306102 (PMC11207121; doi:10.1371/journal.pone.0306102)
Supplement: S1 Table — (DOCX) [file pone.0306102.s001.docx]

**S1 Interview guide**

**Present life with the illness**

I am interested in how you are currently living with ALS and dealing with the illness. Could you tell me about it?

(e.g., family/social environment/occupation/support-group)

**Past illness experiences**

What were your thoughts after you got the diagnosis?

What experiences have you had with support and advice from physicians, nurses and therapists?

Is there anything you would prefer to be changed or done differently?

(e.g., additional information, setting)

**Future plans**

What are your thoughts about your future?

What do you think about future treatment and care?

**Informational and counselling needs for life-sustaining treatments, decision-making and options regarding death and dying**

Some PALS opt for life-sustaining treatments such as a PEG or ventilation; others do not. What do you think about these options?

Options:

- PEG, NIV, TIV
- Rejection and discontinuation of treatments
- Palliative care and hospice
- Advance decision-making and advance directive

What information have you received about life-sustaining options?

(If applicable, how/where/by whom?)

Have you searched for information by yourself?

How satisfied were you with the timing of the counselling?

How were you informed about communication loss and communication support/aids?

Have your decisions/attitudes concerning life-sustaining treatments changed over time?

Do you have any wishes on how counselling/support should be done differently?

What (additional) support would you like to have in making decisions about these options?

Some people wish to end their life prematurely. What do you think about this?*

*If necessary, clarification of the non-permissibility of euthanasia in Germany or differentiation of euthanasia / assisted suicide and legal classification.

If yes: Have you ever thought about travelling to Switzerland (for assisted suicide)?

Do you think it makes a difference whether the person concerned takes the drug themselves or whether someone else administers it to them (e.g. the physician gives them an injection)?

Some people voluntarily stop eating and drinking to bring about their death more quickly. What do you think about it?

When a dying person suffers severely, physicians can give them medication that helps them sleep permanently and no longer notice the symptoms (palliative sedation). What do you think about it? Would this be an option for you?

Have you sought advice or information on these topics? What were the reactions? What reaction/help would you have wished for?

What do you think about end-of-life care for PALS in Germany and options to decide how you want to die?

**Ending the interview**

What is particularly important to you in life? What gives you the strength/motivation to live?

Is there anything we have not talked about yet that you would like to share?

How are you feeling now after this conversation? Is there anything I can do for you?

**Abbreviations**

ALS: amyotrophic lateral sclerosis

PALS: people with ALS

PEG: percutaneous endoscopic gastrostomy

NIV: non-invasive ventilation

TIV: tracheostomy with invasive ventilation
